# Impact of Job Resources and Job Demands on Burnout among Physical Therapy Providers

**DOI:** 10.3390/ijerph182312521

**Published:** 2021-11-28

**Authors:** Rupal M. Patel, John Bartholomew

**Affiliations:** Department of Kinesiology and Health Education, University of Texas at Austin, Austin, TX 78757, USA; jbart@austin.utexas.edu

**Keywords:** occupational health, burnout, physiotherapist, physical therapist, self-determination theory, job resources–demands

## Abstract

Job burnout is a threat for physical therapists. Little research has been conducted to identify possible protective factors against burnout in this population. Accordingly, we utilized the job demands-resources (JD-R) model and self-determination theory to guide our examination of basic psychological needs as factors to predict burnout in physical therapists. One hundred and two licensed physical therapists completed surveys. Higher levels of autonomy, competence and relatedness predicted burnout, even after accounting for job demands. Job resources, such as the basic psychological needs outlined by the self-determination theory, along with perceived respect, buffer the negative effects of secondary trauma on burnout. The job resource of perceived respect also buffers the negative effects of the physical demands of the job on burnout. These findings suggest that while job demands can be difficult to change, increasing the fulfillment of job resources can help ameliorate burnout in physical therapists. The present findings highlight the need for workplace interventions which cultivate the satisfaction of the basic psychological needs of autonomy, competence, and relatedness to address burnout among physical therapists.

## 1. Introduction

There is a concern regarding burnout among healthcare professionals [1]. Burnout is a form of occupational stress that manifests over time and includes the following symptoms: feelings of cynicism (i.e., inability to connect to clients resulting from negative or cynical feelings about them), overwhelming exhaustion (i.e., depletion of psychological resources) and a lack of accomplishment [2,3]. It has long been recognized that burnout disproportionally affects individuals who spend greater amounts of time in direct patient care [4,5]. This is especially the case for physical therapists (PTs) whose primary responsibilities involve the direct care of patients suffering from musculoskeletal ailments. 

The negative effects of burnout can be devastating for the patient, practitioner, and organization. Practitioner burnout and well-being are significantly associated with the safety of the patient, as measured by self-reported errors [6], a relationship that appears cyclical as self-reported errors have also been associated with increased burnout and depression [7]. There are also numerous personal consequences of burnout, including depression, alcohol abuse [7], musculoskeletal pain and an increased risk of cardiovascular disease [8]. Organizations also suffer from employee burnout due to the increasing costs of employee turnover [1], patient errors and lower overall patient satisfaction [9]. Despite the deleterious effects of burnout on the provider, organization and field, there has been limited progress in reducing burnout in this population. Thus, a primary objective of this study was to identify factors that may protect against burnout in PTs and therefore provide guidance for future interventions.

To provide a theoretical basis for our work, we utilized the job demands-resources model [10] to guide our basic understanding of job burnout. The job demands-resources (JD-R) model [11] describes work stress through two categories: job demands and job resources [11,12,13]. Job demands include the psychological, physical or organizational aspects of one’s job that exceed the capabilities of the employee, create stress and lead to burnout over time [14]. Job resources are psychological, physical or organizational aspects of a job that function to achieve work goals, reduce job demands and stimulate personal growth [14]. While job demands are recognized as a necessary part of every job, they can become problematic if they are not adequately balanced with job resources. This imbalance creates job stress that, if unaltered, is likely to lead to burnout [10]. 

The JD-R model provides an empirically supported theoretical model for understanding job burnout, but it does not suggest which factors are specifically resourceful or demanding. As such, this study utilized self-determination theory (SDT) to identify innate human needs that are essential for psychological well-being. 

Self-determination theory (SDT) [15,16] postulates that three basic psychological needs must be met in order for an individual to thrive in a work environment: (1) autonomy, (2) competence and (3) relatedness [17]. According to Maslow’s Hierarchy of Needs, a job that satisfies the higher-level needs of an individual worker will determine its ability to motivate and increase a person’s affective experience [18]. The need for autonomy includes the psychological freedom to carry out an activity of one’s own volition. At work, this includes being able to connect with the importance of their work, for example, feeling valued for their professional opinion or having independence when executing a task [19]. Autonomy has been associated with less burnout, particularly emotional exhaustion [19]. Donohoe et al. found that 46% of PTs working in rehabilitation settings (*n* = 129) experienced moderate burnout, with the greatest predictor being emotional exhaustion [20]. According to Maslach, emotional exhaustion is a lost sense of accomplishment that prompts the individual to create an emotional and cognitive distance between themselves and their work [21]. Wolfe describes this as a primary antecedent to burnout, stating that this is particularly problematic for physical therapists as the natural expectation between a patient and therapist is the alleviation of some presenting problem [22]. If these expectations are not met, the therapist may experience failure and frustration that can lead to personal inadequacy manifesting as self-anger, especially when it occurs within a low-autonomy environment [22].

The second need, competence, involves an individual’s need to feel effective in their chosen field, which is directly related to job resources and stress. Balogun found that 58% of physical therapists and occupational therapists reported high levels of burnout (*n* = 187) [23]. This study found that the physical and emotional well-being of PTs and occupational therapists was impacted by uncertainty in the organizational climate and job stress [24]. Job stress in these rehabilitation professionals has been defined as the incongruency between institutional demands (e.g., high productivity standards and paperwork) and the personal and professional values of the provider (e.g., the impact they are able to have on the lives of their clients) [24]. Additionally, Wolfe describes the inability to reach desired goals in the work environment as “work overload” [22]. This not only includes the overload that occurs when a facility is understaffed or has high productivity standards, but also having insufficient skills. This also includes the emotional fatigue that stems from a therapists’ inability to apply the full use of their therapeutic skills due to a lack of time or autonomy [22]. Individuals with a higher skillset and knowledge in this context have demonstrated a greater incidence of burnout [25].

Relatedness is based on the need to feel accepted and cared for in relationships with others. Work environments where a team model of treatment is valued and positive interpersonal relationships are maintained help fulfill this need. However, physical therapists often find themselves in situations where decisions are ultimately made by physicians while ignoring the input of the therapist. This can lead to role ambiguity, which has been associated with emotional exhaustion in PTs [24]. Role ambiguity is the frustration that may arise in response to unclear expectations regarding authority [24].

Van den Broeck and colleagues (2010) found that the fulfillment of these basic psychological needs is positively associated with general and psychological well-being. The satisfaction of these basic needs has also been found to be protective against burnout [26,27,28]. These promising findings have yet to be tested in a sample of physical therapists. The consideration of these basic psychological needs in the workplace can help to counter the negative effects of job demands and lessen the symptoms of burnout. Additional job demands considered in this setting include the physical demands of the job and accumulative traumatic stress that manifests in the face of working with victims of trauma.

A further source of stress is the physical demands of the job. PTs are often engaged in high-force tasks which put them in compromising positions throughout the day. Patient handling, such as transfers and lifting, has been associated with work-related musculoskeletal disorders and has demonstrated high biomechanical loads [29]. One would expect their experience of their career to be impacted by their own physical health. A recent systematic review found that up to 90% of PTs experience work-related musculoskeletal disorders in their careers [30]. Research demonstrates that the association between musculoskeletal disorders and burnout varies with occupation [31]; however, to our knowledge, no studies have tested these effects in physical therapists. Given the considerable physical load inherent in the job, and the subsequent demands it places on the therapist [32], it is not unreasonable to assume that physical therapists would be especially vulnerable to increased burnout due to their own challenges with physical functioning.

Additionally, physical therapists often provide support for individuals who have undergone some form of trauma. This level of caregiving requires additional emotional resources and has been associated with psychological distress [33]. Compassion fatigue (CF) occurs when a caregiver exhibits a reduced capacity for empathy towards their patient [33]. The two components of CF include secondary traumatic stress and burnout. Secondary traumatic stress is the manifestation of stress [34] in response to a secondary exposure to traumatic events [35,36]. This includes listening to descriptions of events that have led to physical and emotional injury in a patient. There is some overlap between secondary trauma and burnout; however, burnout is due to a long-term exposure to work-related stressors while STS can take effect after listening to a single traumatic account [33,36].

The literature suggests that the professional quality of life of physical therapists is associated with their perceived impact on their patients [22,24], ability to apply their skillset adequately [22], and the respect they receive as a professional [23]. Additionally, increased job demand in this population has been associated with work-related musculoskeletal disorders [29,32,37] and secondary traumatic stress [38]. Accordingly, we developed a questionnaire that reflects the extant literature, with an emphasis on perceived impact, perceived respect, physical demands of the job and skillset. This reflects the core concepts of job satisfaction as stated by Hulin and Judge (2003), including the fact that the psychological responses to one’s job have evaluative, emotional and behavioral components [19,39]. 

To the best of our knowledge, there is limited research on factors that protect or reduce burnout in PTs. Accordingly, the present study will address this gap in the literature by applying a well-researched framework to better understand job burnout in PTs. As such, our hypotheses are as follows: (1) greater PT burnout will be associated with greater job demands and lower job resources; (2) all job resources (autonomy, competence, relatedness, impact, respect, and skillset) will buffer the positive relationship of each of the two job demands (physical demands and secondary trauma).

## 2. Materials and Methods

We conducted an on-line, cross-sectional survey of physical therapists who are licensed to practice in the state of Texas to determine their level of burnout and associated factors. This was created on Qualtrics (Qualtrics, Provo UT USA), an online reporting software that could be completed on a computer-, tablet- or phone-based browser. As this study used human participants, ethical approval was obtained from the Institutional Review Board for Human Subject Research at the University of Texas at Austin. The potential sample was based on those PTs who provided a valid email address through the American Physical Therapy Association (APTA) and had internet access. In line with email survey responses and other PT survey studies, the authors aimed for a response rate of 25% [40,41]. The principal investigator reached out to the 511 valid email addresses in early November 2019. The email explained the nature of our study and the procedures for consent. Once consent was provided, participants then used an anonymous link to access the survey on the Qualtrics platform. A reminder email was sent the following week to recruit additional participants and thank the ones who had already completed the survey. The survey was closed on the Qualtrics platform in late November 2019.

### 2.1. Data Analysis

Data were analyzed using SPSS statistical software (IBM Corp, Armonk, IL USA). Analyses began with descriptive statistics followed by a series of categorical comparisons and a hierarchical multiple regression analysis with burnout as the dependent variable. Eleven total models were examined. In the first model, relevant demographic variables were entered in the first step, followed by job demands and resources in the following steps: job demands of secondary trauma and physical demand (step 2), the job resources of autonomy, competence and relatedness (step 3), and the job resources of impact and respect (step 4). Additionally, burnout was regressed on age, total debt and setting in step one, job demand (either secondary trauma or physical demand) in step 2, job resource (either autonomy, competence, relatedness, impact or respect) in step 3 and a two-way interaction (job resource x job demand) in step 4 to assess if any job resource moderated the relationship between job demand and burnout. Supported by the literature on the subjects necessary for an adequately powered regression analysis [42], a post hoc power analysis (conducted in G*Power 3.1) revealed that 74 subjects were needed to achieve a power of 0.95 at an alpha level of 0.05 and effect size of 0.15. 

### 2.2. Measures

#### 2.2.1. Demographic Data 

Participants were asked to report their gender, year of birth, education level, income, zip code, employment status, number of co-workers, current debt from PT school loans, total debt from PT school loans, work setting, specialty, title, year they graduated from PT school, hours worked per week and percentage of time in direct patient care. 

#### 2.2.2. Work Burnout Scale 

The Work Burnout Scale is an 8-item scale used to measure degree of job burnout and well-being of professionals [33]. The Work Burnout Scale was originally part of a 13-item scale to measure compassion fatigue (Compassion Fatigue-Short Scale) but demonstrates adequate psychometric properties to be used as an individual scale [33]. The Work Burnout Scale presents respondents with items such as “I have felt trapped by my work” and asks them to rate their level of agreement on a 7-point, Likert-type scale, with 1 indicating “not true at all” and 7 indicating “very true”. The scores were summed, with higher scores being indicative of higher levels of job burnout. The scale has demonstrated a good internal reliability with a Cronbach’s alpha of 0.90 in previous research [33] and, in this study, a Cronbach’s alpha of 0.85. 

#### 2.2.3. Secondary Trauma Scale 

The Secondary Trauma Scale is a 5-item scale used to measure the degree of vicarious trauma related to client interactions [33]. The Secondary Trauma Scale was originally part of a 13-item scale to measure compassion fatigue (Compassion Fatigue-Short Scale) but demonstrates adequate psychometric properties to be used as an individual scale [33]. The Secondary Trauma Scale presents respondents with items such as “I have flashbacks connected to my clients” and asks them to rate their level of agreement on a 7-point, Likert-type scale, with 1 indicating “not true at all” and 7 indicating “very true”. The scores were summed, with higher scores being indicative of higher levels of secondary trauma. The scale has demonstrated good internal reliability with a Cronbach’s alpha of 0.80 in previous research [33] and, in this study, a Cronbach’s alpha of 0.81. 

#### 2.2.4. Basic Psychological Needs at Work Scale 

The Basic Psychological Needs at Work Scale [43,44,45] is a 21-item scale used to measure three basic psychological needs: autonomy, competence and relatedness. Each of these are assumed to be universal and innate according to self-determination theory [16]. Autonomy (e.g., “I feel like I can make a lot of inputs to deciding how my job gets done”), competence (e.g., “People at work tell me I am good at what I do”) and relatedness (e.g., “I really like the people I work with”) were assessed. Respondents were asked to rate their level of agreement on a 7-point Likert-type scale, with 1 indicating “not true at all” and 7 indicating “very true”. The responses were averaged for each dimension, with higher scores being indicative of higher levels of fulfillment. The scale showed acceptable internal reliability in our study, with a Cronbach’s α of 0.76 for autonomy, 0.67 for competence, and 0.83 for relatedness.

#### 2.2.5. Physical Therapy Satisfaction Scale 

The Physical Therapy Job Satisfaction Scale (PTSS) was developed for this study to assess field-specific aspects of job satisfaction of physical therapists. After reviewing existing scales in other fields and discussion with active physical therapists, we developed 25 items that were scored on a 7-point Likert-type scale, with 1 indicating “not true at all” and 7 indicating “very true”. All 25 items were given to our participants with responses examined through an exploratory factor analysis. Varimax rotation was used as these were likely to be correlated factors. This resulted in three factors. Within these factors, items were eliminated based on: having an eigenvalue factor score of 0.40 or greater on two or more components or having a communality of less than 0.50. This resulted in a final 11-item scale with 3 sub-scales. The physical demands subscale is composed of 4 items that are designed to assess the physical demands of the respondents’ jobs (e.g., “At the end of the workday, I experience physical pain”). This demonstrated acceptable reliability in our sample—Cronbach’s α = 0.81. The impact subscale is composed of 4 items that are designed to assess the PTs perception of the impact their treatment has on their patients (e.g., “I am confident that my patients have lasting effects from my treatment”). This demonstrated acceptable reliability in our sample—Cronbach’s α = 0.82. The respect sub-scale is composed of 3 items that are designed to assess how respected they feel at their place of work (e.g., “My professional opinion is often overruled by others”). This demonstrated acceptable reliability in our sample—Cronbach’s α = 0.79. The sum of each sub-scale was taken to reach the total score, with higher scores being indicative of greater fulfillment in the given domain. As this is a newly developed scale, all items are presented in Table 1. 

## 3. Results

### 3.1. Sample Demographics 

Of the 511 licensed physical therapists in Texas who were emailed the survey, 450 were valid addresses (61 were returned for being invalid). Of these 450, 118 completed the questionnaire, a 26.2% response rate. Sixteen responses were incomplete and eliminated from the analysis. Table 2 gives the demographic characteristics of the respondents. The majority were female (69.2%), with an average age of 44 years old (range: 26–69) and a median income of USD 70,000–79,000. These data are consistent with the national averages, with 69.5% females in the PT workforce, an average PT salary of USD 72,820.00 and mean age of 41 years (DataUSA, n.d.). The total amount of debt incurred during PT school was less than ten thousand dollars for 40.7% of respondents, with 11.7% reporting a value greater than USD 100,000. The majority reported working in a private practice setting (41.1%) followed by the hospital setting (28.9%). The majority reported working in orthopedics (37.4%) and held the title of Senior or Head PT. Fifty-seven percent reported having less than 10 years’ experience as a practicing PT and 65.7% reported spending between 71 and 100 percent of their day in direct patient care. 

### 3.2. Preliminary Analyses 

Table 3 presents the means, standard deviations, sample sizes, internal consistencies and correlations between the measures. An examination of the correlations between the demographic variables and burnout revealed a significant association between age and burnout (*r* = −0.27, *p* = 0.007) and total debt burden (*r* = 0.31, *p* = 0.002). Additionally, previous research found that burnout differs by therapeutic setting [46]. Thus, these demographic variables were entered in the first step of the regression analysis. The remainder of the demographic variables were not included in further analysis as none were found to be significantly correlated with burnout.

### 3.3. Impact of Covariates on Burnout

To examine the direct impact of the covariates of age, total debt and setting on burnout, they were entered in step one of our model. Total debt (*β* = 0.24, *p* =0.02) had a significant association with burnout. Age (*β* =−0.19, *p* = 0.07) and setting (*β* = 0.14, *p* = 0.16) did not have a significant association with burnout. 

### 3.4. Impact of Job Demands on Burnout 

Analyses were grouped as a function of the category for job demands: (1) secondary trauma and (2) physical demands and their impact on burnout. After controlling for age, total debt and therapeutic setting in step 1, secondary trauma and physical demand (job demands) predicted significantly higher rates of burnout (*β* = 0.42, *p* = 0.000, *β* = 0.34, *p* = 0.000, respectively) and accounted for a significant proportion of the variance [F_change_ (2, 94) = 35.50, *p* < 0.000]. Together, these job demands accounted for 51% of additional variance in burnout in this model.

### 3.5. Impact of Job Resources on Burnout

Analyses were grouped as a function of the category for job resources: (1) autonomy, (2) competence, (3) relatedness, (4) impact and (5) respect and their impact on burnout. Step 3 added the job resources of autonomy, competence, and relatedness. Autonomy and competence were found to be significant predictors of burnout (*β* = −0.24, *p* = 0.003, *β* = −0.32, *p* = 0.000, respectively). Relatedness was not found to be a significant predictor of burnout (*β* = 0.13, *p* = 0.10). The addition of these predictors accounted for a significant, unique portion of the variance [F_change_ (3, 91) = 13.81, *p* < 0.000]. The predictors in Step 3 accounted for 15% of the unique variance in burnout. Step 4 included the addition of the physical therapy-specific job resource variables developed for this study: respect and impact. The results demonstrated no significant change in the accounted for variance, [F_change_ (2, 89) = 0.55, *p* > 0.15, R^2^_change_ = 0.00].

### 3.6. Interaction between Job Demands and Job Resources on Burnout 

**Figure 1:**
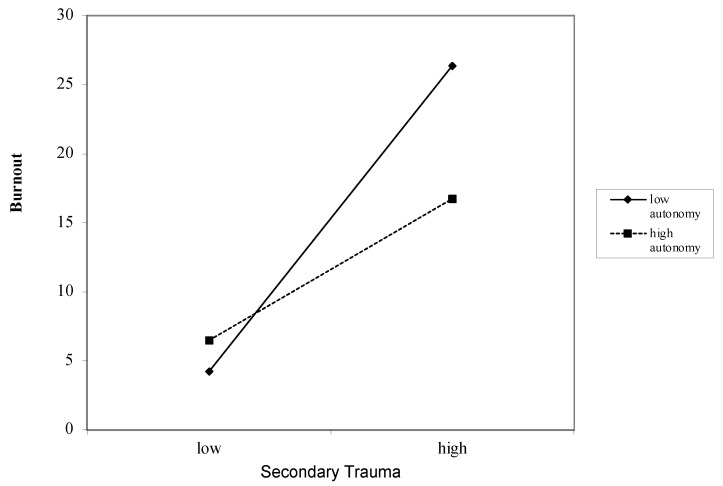
Graph of interaction plot between secondary trauma and autonomy. Perceived autonomy moderates the relationship between secondary trauma and burnout, such that those with low autonomy (1 SD below the mean) and high secondary trauma reported greater burnout.

**Figure 2:**
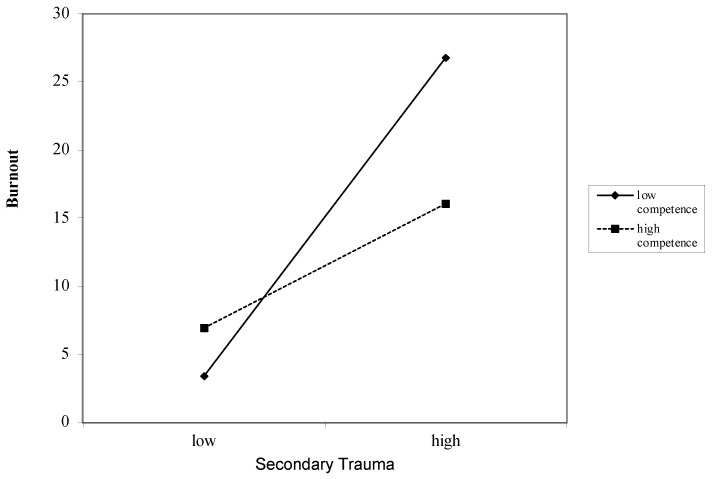
Graph of interaction plot between secondary trauma and competence. Perceived competence moderates the relationship between secondary trauma and burnout, such that those with low competence (1 SD below the mean) and high secondary trauma reported greater burnout.

**Figure 3:**
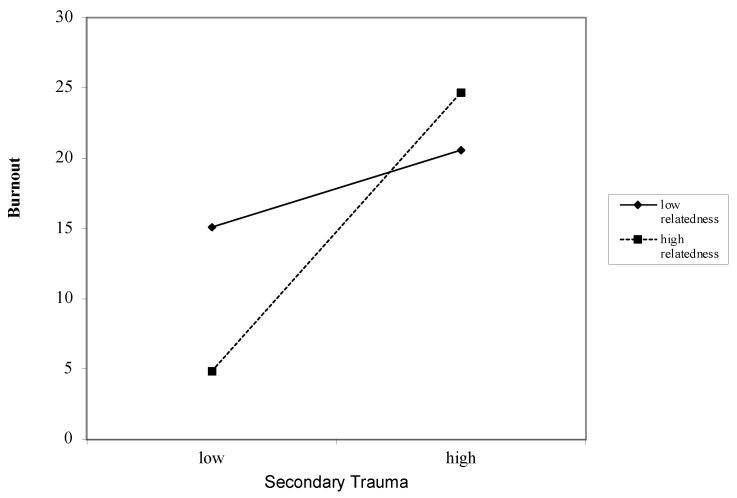
Graph of interaction plot between secondary trauma and relatedness. Perceived relatedness moderates the relationship between secondary trauma and burnout, such that those with low relatedness (1 SD below the mean) and high secondary trauma reported greater burnout.

**Figure 4:**
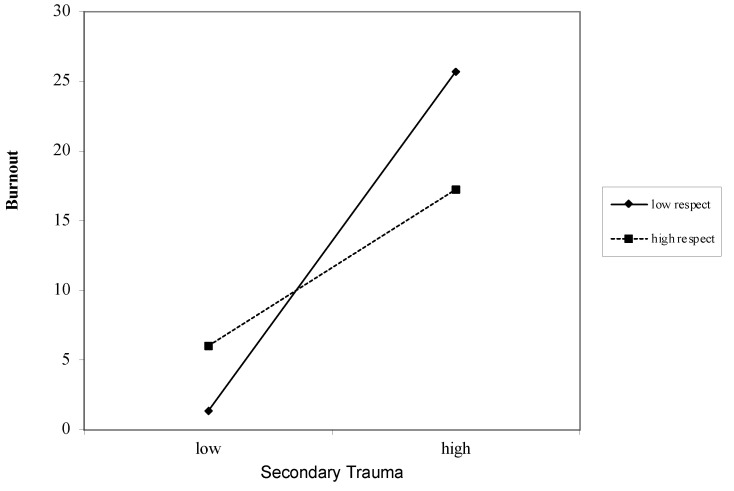
Graph of interaction plot between secondary trauma and respect. Perceived respect moderates the relationship between secondary trauma and burnout, such that those with low respect (1 SD below the mean) and high secondary trauma reported greater burnout.

Secondary Trauma as a Job Demand for Burnout (Table 4): Overall, secondary trauma was associated with burnout after accounting for covariates (*β* = 0.57, *p* < 0.000). These associations remained after including the job resources of autonomy (*β* = 0.50, *p* < 0.000), competence (*β* = 0.50, *p* < 0.000), relatedness (*β* = 0.54, *p* < 0.000), impact (*β* = 0.55, *p* < 0.000), and respect (*β* = −0.26, *p* < 0.000). Interactions between secondary trauma and the job resources of autonomy (*β* = −1.01, *p* < 0.000) (Figure 1), competence (*β* = −1.70, *p* < 0.000) (Figure 2), relatedness (*β* = 2.74, *p* < 0.000) (Figure 3), and respect (*β* = 0.55, *p* < 0.000) (Figure 4) were found to be significant. The interaction between secondary trauma and the job resource of impact was not found to be significant (*β* = −0.65, *p* = 0.50).

**Figure 5:**
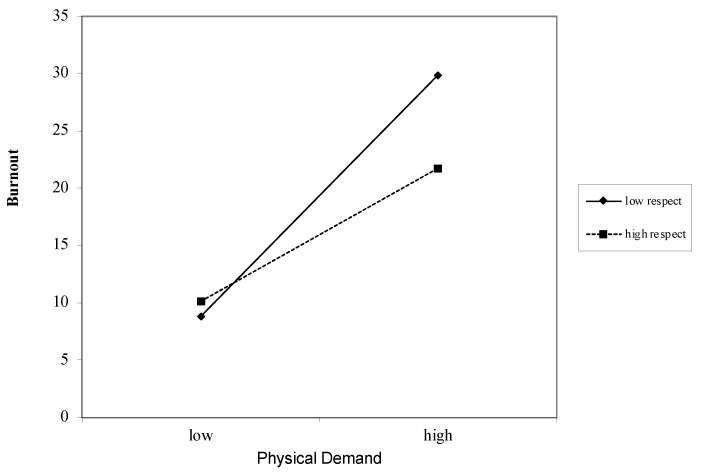
Graph of interaction plot between physical demands and respect. Perceived respect moderates the relationship between physical demand and burnout, such that those with low respect (1 SD below the mean) and high physical demand reported greater burnout.

Physical Demands as a Job Demand for Burnout (Table 5): Overall, physical demands were strongly associated with burnout (*β* = 0.50, *p* < 0.000). These associations remained after including the job resources of autonomy (*β* = −0.40, *p* < 0.000), competence (*β* = 0.44, *p* < 0.000), relatedness (*β* = 0.48, *p* < 0.000), impact (*β* = 0.50, *p* < 0.000), and respect (*β* = 0.50, *p* < 0.000). The interactions between physical demands and the job resource of respect (Figure 5) were found to be significant (*β* = −0.95, *p* =.05) (Figure 5). The interactions between physical demands and the other job resources were not found to be significant: autonomy (*β* = −0.64, *p* = 0.12), competence (*β* = −0.53, *p* = 0.31), relatedness (*β* = −0.56, *p* < = 0.32), and impact (*β* = 0.06, *p* = 0.94).

## 4. Discussion

This is the first study to examine the role of basic psychological needs in burnout among physical therapy providers. Our findings suggest that the job demands (secondary trauma and physical demand) and various job resources (autonomy, competence, relatedness, impact and respect) we tested have a significant association with burnout in this population. As such, our hypothesis that greater burnout would be associated with greater job demands and lower job resources was supported. Additionally, our hypothesis that all job resources would buffer the positive relationship of each of the two job demands was partially supported. 

As laid out in the JD-R model, stress and burnout are the result of the balance between job demands and job resources. Our data supported this model. The job demands of secondary trauma and physical demands were positively correlated with burnout. Additionally, the five job resource factors of autonomy, competence, relatedness, respect and impact were negatively correlated with self-ratings of burnout. This is consistent with research in other fields that have found significant positive associations between job demands and burnout and negative associations with job resources and burnout [14,47]. Consistent with self-determination theory, all three basic needs (autonomy, competence, and relatedness) were found to be negatively correlated to burnout in our sample. This, again, is consistent with the extant literature and has been observed cross-culturally as well as across various life domains and work disciplines [13]. 

After controlling for significant demographic variables, we found secondary trauma and physical demands (*job demands*) to have a statistically significant contribution to job burnout. This finding is consistent with the JD-R model, which recognizes that job demands can assume physiological and psychological costs, especially when working in high-demand situations that require close patient interactions [14]. In testing the protective effect of the basic psychological needs outlined by self-determination theory, we found all three to be negatively associated with burnout. Consistent with our findings, Bakker et.al. (2005) showed that employees who experienced higher levels of fulfillment of psychological needs were protected against burnout due to increased job demands. The development of autonomy in one’s work, competence in clinical skills and positive relations within the team in the clinical context should, therefore, serve to protect against burnout in this sample. Additionally, the existing literature demonstrates that the fulfillment of these innate psychological needs provides a buffer against high job demands [47]. Our findings are consistent with previous work positing that job resources provide a protective buffer against job demands. Of the five tested moderators, four demonstrated a protective effect against secondary trauma and one against physical demands. PTs reporting high levels of burnout and secondary trauma had low levels of autonomy, competence, relatedness and respect. PTs reporting high levels of burnout and physical demands had low scores in the respect category of job satisfaction. It is interesting to note that perceptions of impact did not buffer the effect of secondary trauma on burnout; however, this may be due to the shared variance with the other protective factors. Additionally, it is interesting to note that the perception of respect was the only variable that buffered the effect of physical demands on burnout. This may reflect the differences in therapeutic settings. Our sample consisted of 41% of PTs working in private practice settings, where practitioners may have a greater freedom of choice and may experience less emotional exhaustion stemming from role ambiguity. Schuster and colleagues found that 53% of the PTs surveyed (*n* = 160) experienced burnout [46], with acute care having the highest prevalence of symptoms. Conversely, a greater percentage of individuals in the non-burnout group were in private practice, suggesting that setting may play a role in the symptoms of burnout in this population [46].

It is interesting to note the minimal variance explained by the demographic data. While debt and age were related to self-reports of burnout, the effects were rather small. Job satisfaction and burnout appear to instead be largely driven by the day-to-day perceptions of job demands and one’s resources to manage these demands. It is especially interesting that salary had no relationship with the other variables. We often focus on economic issues when considering job satisfaction and burnout. Our data indicate that these perspectives are misplaced. We must, instead, consider how our systems of practice are designed and the extent to which they provide resources to support what is necessarily a demanding profession. 

### 4.1. Practice Implications

Job demands associated with the practice of physical therapy are unavoidable given the physical nature of clinical services in addition to the rising cost of healthcare coupled with cuts in reimbursement, high productivity standards, and the emotional burden inherent in the job [24,48]. Despite these challenges, it is encouraging to find evidence that the fulfillment of the basic psychological needs might be able to reduce job burnout in this population. 

Interventions to reduce burnout and improve employee well-being have generally focused on individual-level changes rather than the kind of multi-level approaches that may impact systems of practice and thus prove to be more effective [49]. Leadership training has been demonstrated as one of the most cost-effective ways to improve the overall health of an organization and its employees [49]. This training can include manager-led efforts to increase employee autonomy, which have been shown to increase job satisfaction and social support [19]. For example, the introduction of manager-led discussions increases the satisfaction of basic needs (autonomy, competence and relatedness) in employees [50]. Further, increasing self-efficacy among employees has been demonstrated to reduce burnout [51]. Given these data and the results of the present study, organizations might consider offering targeted job trainings and continuing education courses to increase clinical skills and decision making to increase feelings of self-efficacy, competence and autonomy among their workers. 

Protective factors against work-related psychological phenomena including burnout and STS can be developed through mindfulness and resilience-building interventions [52,53]. Given the present finding of 35% of our sample experiencing moderate to high levels of secondary trauma and the relationship between STS and burnout, it would likely benefit organizations to include this form of training to support their PTs in managing the experience of secondary trauma. 

Our initial exploration of PT-specific domains of job satisfaction was consistent with findings in the literature which found that work-related musculoskeletal disorders in physical therapists negatively affect quality of life and job factors [32]. In response, there have been calls for a shift in culture to value the needs of PTs as much as the profession values the needs of their patients [32]. If this is supported by broad training to reduce job demands and improve job resources, our data suggest that these would be associated with longevity for the practice of a PT as well as improve the practitioner’s overall well-being [32]. Additional research in this area is warranted given the breadth and scope of this problem.

### 4.2. Limitations

The limitations of this study include its cross-sectional design. Taking measurements at one point of time prevents any effort to identify causal effects or the direction of the observed relationships. While appropriate given the dearth of data, future studies should consider a longitudinal design to study the predictors of burnout in PTs. Additionally, our sample size and recruitment from a single state prevent the analysis of meaningful sub-groups—especially as states differ in their practice regulations. Increasing the sample of PTs to be both more diverse and from multiple states would improve the ability to generalize these data. Further, our study utilized a novel measure of PT job satisfaction. Face-valid and novel measures are common when working in specialized settings but require additional validation. Our development of this measure included an exploratory factor analysis and the resulting scale demonstrated strong reliability. Despite this, the scale was not validated outside of this sample nor was a confirmatory factor analysis completed. Given these factors, the results should be interpreted with caution. Future research should expand on the efforts to validate this scale.

## 5. Conclusions

Despite these limitations, this study advances our knowledge of the associations between core psychological needs and job burnout in physical therapy providers. These findings are consistent with the extant literature that suggests that the satisfaction of psychological needs reduces burnout, even after taking into account the negative effects of job demands [47]. These findings highlight the need for workplace interventions which cultivate the satisfaction of these basic psychological needs to ameliorate burnout for these healthcare providers. Further, our findings on secondary trauma in this population introduce a new potential avenue for intervention. Future research on burnout in physical therapists should consider utilizing the combined framework of the JD-R and self-determination theories to address burnout in physical therapists.

## Figures and Tables

**Table 1:** Physical Therapy Job Satisfaction Scale.

Physical demand	1	At the end of the workday, I experience physical pain.
	2	I am concerned about the longevity of my career due to the physical strain my job puts on my body.
	3 *	I enjoy the physicality of my work.
	4	The physical nature of my work restricts my ability to have physically active hobbies
	5 *	My quality of life is not impacted by the physical nature of my job.
Skillset	1	I am encouraged to explore my own areas of interest for treatment of my clients
	2	I am able to apply my individual skillset to treat my clients
	3	I have the freedom to dictate my own continuing education development for techniques for treatment of my clients.
	4 *	I feel restricted by the techniques I am allowed to use in my workplace
	5 *	I do not feel adequately prepared to treat the clients who need my help.
	6	I have developed an expertise within Physical therapy that I am proud of.
Impact	1	I feel like the education I provide to patients helps them better their overall health.
	2	I am confident that my patients have lasting effects from my treatment.
	3 *	I do not feel like my treatments provide long term relief to my clients.
	4	Most of my clients see improvements in their symptoms
	5	I make a positive difference in people’s lives
	6 *	The amount of paperwork I am required to do impacts how effective I can be as a clinician.
Respect	1	I am glad this is the profession I chose.
	2	I am proud to tell people what I do for a living
	3	My job title is respected by others in the community.
	4 *	I try to avoid talking to others about my work.
	5	I am treated as an equal by other healthcare professionals.
	6 *	Physicians often talk down to me.
	7 *	My professional opinion is often overruled by others.
	8	My clients appreciate the care I give them.

* reverse coded items.

**Table 2:** Demographic characteristics.

Characteristics	Frequency	Percent *
Gender		
Female	74	69.2
Male	33	30.8
Age		
25–30	17	16.0
31–40	29	27.4
41–50	20	18.9
51–60	25	23.6
61–70	15	14.2
Income		
<USD 50,000	5	4.6
USD 50,000–59,000	4	3.7
USD 60,000–69,000	7	6.6
USD 70,000–79,000	23	18.7
USD 80,000–89,000	10	9.3
USD 90,000–99,000	16	15.0
USD 100,000–149,000	37	34.6
>USD 150,000	5	4.6
Total Debt		
<USD 10,000	42	40.8
USD 10,000–25,000	16	15.5
USD 25,000–50,000	17	16.5
USD 50,000–75,000	12	11.7
USD 75,000–100,000	4	3.9
>$100,000	12	11.7
Setting		
Private Practice	44	41.1
Hospital	31	29.0
Home Health	3	2.8
Other	29	27.1
Specialty		
Orthopedics	40	37.4
Pediatrics	7	6.5
Spine/Neuro	19	6.5
Geriatrics	7	17.8
Other	34	31.8
Title		
Junior PT	5	4.7
Senior PT/Head PT	44	41.1
Manager	33	30.8
Practice Owner	7	16.8
Other	18	6.5
Years Practiced as PT		
<10	61	57.0
11–15	17	15.9
16–20	8	7.5
21–25	7	6.5
26–30	6	5.6
31+	8	7.4
% of day spent in direct patient Care		
0–30%	20	19.0
31–70%	17	16.2
71–100%	68	65.7

* based on number of responses in each category.

**Table 3:** Means, standard deviations, sample sizes, internal consistencies and correlations between the measures.

Measure	M	SD	N	1	2	3	4	5	6	7	8
Age	44.4	12.45	106								
Debt	10,000–25,000	1.18	103								
Income	80,000–89,000	2.04	107								
Setting	2.16	1.23	107								
1. Autonomy	5.34	0.96	105	(0.76)							
2. Competence	6.05	0.78	106	0.49 **	(0.67)						
3. Relatedness	5.92	0.85	105	0.49 **	0.49 **	(0.83)					
4. Physical	11.67	6.01	105	−0.35 **	−0.18 *	−0.20 *	(0.81)				
5. Respect	18.62	2.98	106	0.38 **	0.49 **	−0.00	0.96	(0.82)			
6. Impact	25.03	2.62	104	0.45 **	0.63 **	0.27 **	−0.05	0.32 **	(0.79)		
7. Burnout	18.90	8.72	104	−0.51 **	−0.51 *	−0.22 *	0.50 **	−0.36 **	−0.36 **	(0.85)	
8.Secondary Trauma	10.91	5.94	105	−0.27 **	−0.21 *	−0.16	0.39 **	−0.12	−0.10	−0.62	(0.81)

Internal consistencies are listed in parentheses; * indicates level of significance: *p* ≤ 0.05; ** indicates level of significance: *p* ≤ 0.01.

**Table 4:** Associations between job resources and secondary trauma with regard to burnout.

	Moderators																			
	Autonomy				Competence				Relatedness				Impact				Respect			
Variables	Step 1	Step 2	Step 3	Step 4	Step 1	Step 2	Step 3	Step 4	Step 1	Step 2	Step 3	Step 4	Step 1	Step 2	Step 3	Step 4	Step 1	Step 2	Step 3	Step 4
Age	−0.19	−0.04	0.02 *	0.05	−0.19	−0.04	−0.02	0.02	−0.19	−0.04	−0.07	0.09	−0.19	−0.04	0.02	0.04	−0.19	−0.04	−0.02	−0.01
Total Debt	0.24 *	0.20	0.18	0.15*	0.24 *	0.17	0.14	0.14	0.24 *	0.17	0.17	0.18 *	0.24 *	0.17	0.20 *	0.21 *	0.24 *	0.17	0.12	0.10
Setting	0.14	0.03	−0.09	−0.13	0.14	0.03	−0.04	−0.04	0.14	0.03	−0.03	−0.12	0.14	0.03	−0.00	−0.03	0.14	0.03	0.04	0.06
Secondary Trauma		0.57 **	0.50 **	1.53 **		0.57 **	0.50 **	2.23 *		0.57 **	0.54 **	−2.13 **		0.57 **	0.55 **	1.24		0.57 **	0.55 **	1.84 **
Moderator			−0.40 **	−0.03			−0.40 **	0.02			−0.16	−0.93 **			−0.31 **	−0.19			−0.26 **	0.10
Secondary Trauma x Moderator				−1.01*				−1.70*				2.74 **				−0.70				−1.31 *
F change	5.04 *	44.88 **	27.53 **	7.78 *	5.09 *	45.36 **	29.77 **	6.22 *	5.04 *	44.88 **	3.41	25.75 **	5.00 *	44.40 **	17.38 **	0.42	5.09 *	45.36 **	11.43 **	8.54 *

* indicates level of significance: *p* ≤ 0.05; ** indicates level of significance: *p* ≤ 0.01.

**Table 5:** Associations between job resources and physical demands with regard to burnout.

	Moderators																			
	Autonomy				Competence				Relatedness				Impact				Respect			
Variables	Step 1	Step 2	Step 3	Step 4	Step 1	Step 2	Step 3	Step 4	Step 1	Step 2	Step 3	Step 4	Step 1	Step 2	Step 3	Step 4	Step 1	Step 2	Step 3	Step 4
Age	−0.19	−0.22 *	−0.14	−0.13	−0.19	−0.22 *	−0.18 *	−0.17 *	−0.19	−0.22 *	−0.24 *	−0.22 *	−0.19	−0.22 *	−0.15	−0.16	−0.19	−0.22	−0.20 *	−0.20 *
Total Debt	0.24 *	0.22 *	0.23 *	0.22 *	0.24 *	0.22 *	0.19 *	0.19 *	0.24 *	0.22 *	0.22 *	0.22 *	−0.24 *	0.22 *	0.25 **	0.25 **	0.24 *	0.22 *	0.16	0.15
Setting	0.14	0.06	−0.04	−0.07	0.14	0.61	−0.01	−0.02	0.14	0.61	−0.00	−0.02	0.14	0.06	0.03	−0.03	0.14	0.61 *	0.07	0.07
Physical Demands		0.50 **	−0.40 **	1.05 *		0.50 **	0.44 **	0.96		0.50 **	0.48 **	1.03		0.50 **	0.50 **	0.42		0.50 **	0.50 **	1.40 *
Moderator			−0.40 **	−0.13			−0.40 **	−0.25			−0.17	0.01			−0.32 **	−0.33			−0.30 **	−0.05
Physical Demands x Moderator				−0.64				−0.53				−0.56				−0.06				−1.00 *
F change	5.09 *	37.00 **	17.70 **	2.51	5.09 *	36.98 **	27.56 **	1.06	5.09 *	36.98 **	3.60	1.01	5.04 **	36.60 **	17.10 **	0.01	5.09 **	36.98 **	13.43 **	3.94 *

* indicates level of significance: *p* ≤ 0.05; ** indicates level of significance: *p* ≤ 0.01.

## Data Availability

Data are available on email request.
